# Clinical and ultrasonographic features of abdominal tuberculosis in HIV positive adults in Zambia

**DOI:** 10.1186/1471-2334-9-44

**Published:** 2009-04-17

**Authors:** Edford Sinkala, Sylvia Gray, Isaac Zulu, Victor Mudenda, Lameck Zimba, Sten H Vermund, Francis Drobniewski, Paul Kelly

**Affiliations:** 1 Tropical Gastroenterology & Nutrition group, University of Zambia School of Medicine, Lusaka, Zambia; 2 Harvard Medical School, Boston, MA, USA; 3 University of Alabama at Birmingham, Birmingham, AL, USA; 4 Vanderbilt University School of Medicine, Nashville, TN, USA; 5 Barts & The London School of Medicine, Queen Mary University of London, London, UK; 6 UK Health Protection Agency, London, UK

## Abstract

**Background:**

The diagnosis of abdominal tuberculosis (TB) is difficult, especially so in health care facilities in developing countries where laparoscopy and colonoscopy are rarely available. There is little information on abdominal TB in HIV infection. We estimated the prevalence and clinical features of abdominal (excluding genitourinary) TB in HIV infected adults attending the University Teaching Hospital, Zambia.

**Methods:**

We screened 5,609 medical inpatients, and those with fever, weight loss, and clinical features suggestive of abdominal pathology were evaluated further. A clinical algorithm was used to specify definitive investigations including laparoscopy or colonoscopy, with culture of biopsies and other samples.

**Results:**

Of 140 HIV seropositive patients with these features, 31 patients underwent full evaluation and 22 (71%) had definite or probable abdominal TB. The commonest presenting abdominal features were ascites and persistent tenderness. The commonest ultrasound findings were ascites, para-aortic lymphadenopathy (over 1 cm in size), and hepatomegaly. Abdominal TB was associated with CD4 cell counts over a wide range though 76% had CD4 counts <100 cells/μL.

**Conclusion:**

The clinical manifestations of abdominal TB in our HIV-infected patients resembled the well-established pattern in HIV-uninfected adults. Patients with fever, weight loss, abdominal tenderness, abdominal lymphadenopathy, ascites and/or hepatomegaly in Zambia have a high probability of abdominal TB, irrespective of CD4 cell count.

## Background

The HIV epidemic in sub-Saharan Africa has contributed to an increased incidence of pulmonary tuberculosis (TB), but there is little information on changes in the frequency or clinical manifestations of abdominal TB. The annual risk of developing active TB, when co-infected with HIV, is 20 to 30 times the risk in seronegative individual [1,2]. In Africa, TB is the leading cause of mortality and morbidity in HIV infected patients, causing up to 41% of HIV-related deaths in Zaire [3]. In Zambia, the number of newly diagnosed cases of TB increased from 8,246 (124/100,000) in 1985 to 38,863 (409/100,000) in 1996 and 52,000 (512/100,000) in 2000 [2]. HIV infection is associated with a rise in the proportion of extrapulmonary TB, including pleural, pericardial, meningeal, lymphadenopathic, disseminated, and bone and joint infections. The proportion of extrapulmonary infection increases as the immunosuppression deepens [4,5]. In one estimate, the ratio of extrapulmonary to pulmonary disease increased from 20:80 in early HIV infection to 50:50 in late HIV infection [6]. As intra-abdominal tuberculosis is difficult to diagnose [7], the contribution of abdominal infection may be higher than previous estimates suggest, but there have been few papers which specifically address this important question. Furthermore, the extent to which the manifestations of abdominal tuberculosis are altered by HIV infection is unknown; we are aware of only one paper which addresses this [8].

The difficulty in diagnosing abdominal TB is due to the lack of efficient and sensitive diagnostic tools as well as its variable anatomical location. Symptoms of abdominal TB are insidious and non-specific which can be mistaken for the constitutional symptoms and signs of HIV disease. In resource-limited settings, there are very few diagnostic tools from which to choose. Microscopy is the most rapid diagnostic tool, which in ideal settings can produce same day results, but it is very insensitive, especially in severely immunocompromised individuals [6]. Culture systems are sensitive, but often take up to four weeks to obtain conclusive results even with enhanced culture systems and these approaches to enable more sensitive and rapid TB diagnosis are not yet available in most developing country settings [9]. Stool culture techniques are of limited value due to the high rates of contamination from other microorganisms and to uncertainty about the significance of isolation of faecal mycobacteria [10]. Most importantly, diagnosis of abdominal TB is limited by the invasiveness and expense of the procedures needed to obtain appropriate samples for histology and/or culture. Laparoscopy or laparotomy, colonoscopy and/or percutaneous biopsy of liver or kidneys may all be required, and culture of ascitic fluid, though more accessible, is insensitive (35% culture positive in a series of patients with peritoneal TB) [7,11]. Ultrasound imaging gives very useful, though not definitive, information [12] but it has not been systematically evaluated in HIV infected patients in Africa. This is important as HIV-related lymphadenopathy may alter the predictive value of established ultrasound findings.

In an autopsy study done in the Ivory Coast to study the causes of wasting, 44% of wasted HIV positive patients who died had TB [13], indicating that weight loss suggests TB in HIV-infected Africans. The other common clinical features of abdominal TB are: fever, abdominal pain, diarrhoea, ascites, hepatomegaly, and splenomegaly [6,7,14,15]. Due to the indolent course of the disease these symptoms often continue for a prolonged period of time before a diagnosis is reached [7]. A heightened clinical suspicion is important because only 15–20% of abdominal TB patients have concomitant active pulmonary tuberculosis [16].

The aims of our study were to obtain a preliminary estimate of the prevalence of abdominal TB in symptomatic HIV-infected patients in the principal in-patient facility in Zambia, assess the level of immunodeficiency in those with abdominal TB, and identify useful diagnostic tools (particularly ultrasound) for a resource poor setting. Based on previous studies of extrapulmonary TB, we decided that HIV infected patients with fever (or night sweats) and weight loss, together with symptoms or signs indicating abdominal pathology, had a sufficiently high clinical suspicion to warrant further evaluation. We used laparoscopy and colonoscopy as gold standard diagnostic techniques, coupled with mycobacterial culture on liquid culture media for the diagnosis of abdominal TB, as these permit definitive biopsy and visual accuracy can be up to 95% [17]. With these as reference standards, we set out to evaluate diagnostic markers which might assist diagnosis in other resource-poor hospitals.

## Methods

### Study population

**Figure 1:**
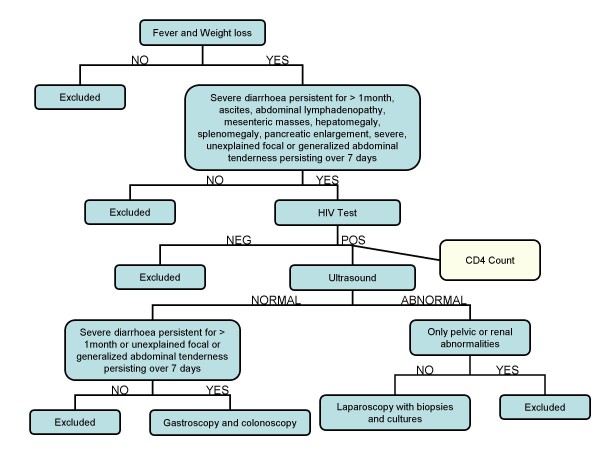
**Diagnostic algorithm for evaluation of patients fulfilling inclusion criteria**.

The 1,200 bed University Teaching Hospital (UTH) in Lusaka functions as the secondary and tertiary care hospital in the city as well as the major referral centre for all of Zambia. Over a period of 27 weeks, from September 2005 until May 2006, all patients admitted to the medical wards of UTH were reviewed by conducting twice weekly ward rounds specifically for this purpose. The inclusion criteria were fever and weight loss, with one or more of the following: diarrhoea persisting for >1 month, ascites, abdominal lymphadenopathy based on ultrasound, mesenteric masses based on ultrasound, hepatomegaly or splenomegaly, pancreatic enlargement based on ultrasound [18] or severe, unexplained focal or generalized abdominal tenderness persisting for 7 days or more. Assessment of weight loss was subjective only, based upon the patient's recollection. A detailed clinical history, physical examination, and laboratory investigations (see below) were carried out, and an abdominal ultrasound was conducted by the study team if not already done or to confirm previous findings. The following were used as exclusion criteria: HIV seronegative, solely pelvic or renal abnormalities, anti-tuberculosis treatment for >1 week, or too sick to undergo laparoscopy or colonoscopy. The decision whether to perform laparoscopy or colonoscopy was based on clinical and ultrasound findings (Figure 1).

HIV seropositive controls were recruited for the purpose of evaluating the specificity of ultrasound findings (especially lymphadenopathy). These were recruited from the HIV clinic at UTH, but they were excluded if they had any of the inclusion criteria suggesting pathology mentioned above within the previous month, any abdominal pathology, or if they had ever had TB.

Ethical approval for the study was given by the University of Zambia Research Ethics Committee and the University of Alabama at Birmingham, Alabama. Informed consent was obtained from all subjects. An information sheet was given to each participant in both English and the predominant local language, Nyanja. Participants were able to withdraw from the study upon request at any time. All patients with definite or probable abdominal TB were treated with an eight month regimen consisting of rifampicin, isoniazid, pyrazinamide and ethambutol as per national guidelines. Anti-retroviral therapy was made available to all participants in this study who required it.

### Definitions

For our study, abdominal tuberculosis was defined as TB affecting the peritoneum, abdominal lymph nodes, omentum, liver, spleen, and/or gastrointestinal tract. A definitive diagnosis of TB was made by demonstration of *Mycobacterium tuberculosis *infection via positive bacteriological culture *and/or *granulomatous inflammation on histopathological examination with positive Ziehl-Neelsen (ZN) staining on microscopy. A presumptive diagnosis of TB was made when granulomatous inflammation was seen on microscopy, or when visual inspection on laparoscopy was consistent with TB and the patient's clinical response to anti-tuberculous treatment was good. Laparoscopic features felt to be consistent with TB for the purpose of making a presumptive diagnosis were the presence of tubercles, fibroadhesive peritonitis, or caseating lymphadenopathy. Diarrhoea was defined as the passage of loose or watery stools more than 3 times in 24 hours.

### Collection of biopsies for culture

Laparoscopies were carried out under general anaesthesia. Two or three 10 mm and 5 mm ports were used to insert biopsy forceps through which biopsies were taken. One part of each biopsy was fixed in 10% formalin, embedded in paraffin wax, and subjected to histopathological examination, PAS, and ZN staining. The other part was placed in saline and submitted for MGIT culture. Colonoscopy was performed under sedation with an Olympus FG-10 colonoscope and biopsies were taken from any diseased mucosa, or from normal mucosa at the ileo-cecal valve if no abnormality was seen. Pathology seen at colonoscopy was not used as a means of making a presumptive diagnosis; positive microscopy or culture was required before starting presumptive treatment. As endoscopic biopsies are smaller than laparoscopic biopsies, three whole biopsies were collected into formalin for histology and three into sterile saline for culture.

### Laboratory methods and measurements

Biopsies were teased with sterile surgical blades and homogenized in a sterile homogenizer for 15 minutes. The vial was centrifuged at 4,000 *g*. In view of the potential contamination of colonic biopsies, all sediments were decontaminated by washing with NaOH to a final concentration of 2% for 15 minutes. After neutralization with phosphate buffer (pH 6.8), 0.5 ml of the centrifuged sediment was pipetted into a Bactec MGIT 960 system tube containing Enrichment supplement (0.8 ml; MGIT system oleic acid-albumin-dextrose-citric acid) and an antimicrobial supplement (MGIT system PANTA [polymyxin B, nalidixic acid, trimethoprim, and azlocillin]) and incubated as described in the manufacturer's manual. The MGIT system was programmed for 7 weeks incubation at 37°C. Positive samples were removed from the machine and subjected to ZN staining for confirmation of the presence of mycobacteria, but no attempt was made to confirm the species.

For *microscopy *quality control, a known ZN positive control slide was stained simultaneously with the test slide. A similar process was performed for Periodic acid Schiff (PAS) staining. Each batch of MGIT system tubes was controlled for support of growth by using *M. tuberculosis *ATCC 27294, *M. kansasii *ATCC 12478, and *M. fortuitum *ATCC 6841.

Blood was submitted for an HIV test (if the status was not already known), CD4 count (FACScount, Becton Dickinson), full blood count, and liver function tests. Ascitic fluid was tapped from those with ultrasound proven ascites and the specimen was centrifuged at 4,000 *g*. Sediments were washed and prepared as above.

### Statistical analysis

Fisher's exact test or the Student's t-test were used to determine statistical significance of associations, as appropriate. The various findings on ultrasound were compared to the final biopsy result of tuberculosis and were classified as true positives (T*p*), true negatives (T*n*), false positives (F*p*), and false negative (F*n*). Positive predictive value (PPV) was calculated as T*p*/total number of positives and negative predictive value (NPV) was calculated as T*n *obtained/total number of negatives.

## Results

During the study period, 5609 patients were reviewed and evaluated. Of these, 140 fitted the main inclusion criteria. We excluded 109 according to the previously mentioned criteria (35 had been on antituberculosis therapy for more than a week, 30 were too sick, 25 were discharged, 8 died without autopsy, 7 declined to participate, and 4 did not return for surgery). The study population, therefore, consisted of 31 patients (Table 1). The age range was 18–54 years (mean 33.1 years). All patients were black Zambians.

**Table 1:** Baseline characteristics, CD4 counts and haematology with respect to final diagnosis

	All patients (n = 31)	Evidence of TB (n = 22)	No evidence of TB (n = 9)	*P*
Sex (M:F)	8:23	7:15	1:8	0.38
Age (mean, SD)	33.4 (8.3)	30.7 (6.9)	39.8 (8)	0.003
Achieved secondary education	11 of 28 known	8 of 20 known	3 of 8 known	0.91
*Clinical features*				
Abdominal tenderness	28 (90%)	19(86%)	9 (100%)	0.53
Generalized lymphadenopathy	12 (39%	9 (41%)	3 (33%)	1.00
Diarrhoea	11 (35%)	8 (36%)	3 (33%)	1.00
Night sweats	11 (35%)	8 (36%)	3 (33%)	1.00
Pallor	10 (32%)	5 (23%)	5 (55%)	0.10
Jaundice	6 (19%)	3 (14%)	3 (33%)	0.32
*Haematological and immunological features*				
CD4 count (mean, SD), cells/μl	125 (128)	92 (115)	194 (134)	0.04
CD4 count below 100 cells/μl	19 (61%)	19 (77%)	2 (22%)	0.02
Haemoglobin concentration (mean, SD) g/dl	9.0 (2.4)	9.8 (2.7)	8.5 (1.7)	0.19
Leucocytes (mean, SD) ×10^9^/l	6.3 (5)	5.1 (2.2)	8.9 (8.1)	0.048
Lymphocytes (mean, SD) %	26.5 (22)	22.5 (16)	34.6 (31)	0.16
Neutrophils (mean, SD) %	66.3 (21)	72.0 (16)	52 (30)	0.02
Platelets (mean, SD) ×10^9^/l	224 (123)	232 (129)	205 (112)	0.59

*P *value refers to the difference between TB positive and negative using Fisher's exact test for proportions or *t *test for continuous variables. Significant differences were found for age, CD4 count and neutrophil count.

Of the 31 fully evaluated patients, laparoscopy was performed on 23, colonoscopy on 5, and a final diagnosis was made at autopsy for 3 patients who died before laparoscopy could be carried out. MGIT cultures were positive in 14 (45%) of 31 patients after anywhere between 21 and 48 days of incubation, and in 2 further patients (6%), microscopy was positive even though cultures failed to grow mycobacteria. In 6 further patients (19%), typical laparoscopic appearances for abdominal TB were seen and the patient responded to anti-tuberculous chemotherapy. Thus, 22 (71%) patients had definite (n = 16) or presumptive (n = 6) abdominal TB. For 14 (45%) of these 31 patients, their HIV infection had not previously been recognized. Three (10%) of TB patients admitted recent contact with another TB person. CD4 counts were lower in patients with evidence of TB (see below) than in patients with no evidence of TB, but other haematological parameters did not differ (Table 1).

The 9 patients with no evidence of TB had the following diagnoses: 2 had diarrhoea of unknown cause, 1 had T cell Non-Hodgkins lymphoma, and 6 had no diagnosis that we could pinpoint. The 2 patients who had diarrhoea presented with fever and weight loss, abdominal tenderness, and loose stools everyday for > 4 weeks; they had 3 stool samples each that showed no organisms. Neither patient had any positive findings consistent with TB on ultrasound and normal colonoscopies. The patient with lymphoma presented with fever, weight loss, diarrhoea for 2 weeks, significant chylous ascites (3 L were drained at laparoscopy), hepatomegaly, mesenteric masses, fibrous stranding throughout the abdomen, and abdominal lymphadenopathy. The 6 patients with no diagnosis presented in a similar manner to the patients with evidence of TB, but biopsies showed non-granulomatous inflammation only and we were unable to confirm response to anti-tuberculous chemotherapy because they were lost to follow-up.

Of the 16 TB patients (52%) who had ascites, 3 patients (19%) had a positive MGIT culture for TB.

### Ultrasound findings

The commonest ultrasonographic finding in the abdominal TB patients was ascites with or without fibrinous stranding. Lymph nodes were also commonly identified: they varied in size from 1.1 cm × 1.8 cm to 2 cm × 3 cm and were found in the para-aortic or porta hepatic region. Table 2 shows the ultrasound findings in the TB positive patients and the PPV and NNV compared with biopsy diagnosis. The ultrasound findings that were most predictive were ascites with fibrinous stranding, hepatomegaly, and mesenteric mass, though none of the findings had a useful negative predictive value.

**Table 2:** Ultrasound findings in relation to evidence of TB

Finding	TB positive	TB negative	PPV	NPV
n = 31	22	9		
Ascites	16	6	73%	33%
Ascites with fibrinous stranding	12	2	86%	41%
Intra-abdominal lymphadenopathy	9	4	69%	27%
Hepatomegaly	8	2	80%	33%
Splenomegaly	1	1	-	-
Mesenteric mass	6	1	86%	33%
Pancreatic mass	0	0	-	-

Note that if some cases of TB were missed through false negative cultures, then PPV and NPV will be under-estimated. Predictive values were not calculated when numbers were very small.

PPV, positive predictive value. NPV, negative predictive value.

None of 28 HIV seropositive controls had ultrasonographically detectable intra-abdominal lymphadenopathy.

### Laparoscopic Findings

The laparoscopic findings varied widely in the 23 patients who underwent the procedure (Table 3). Of these patients, 2 had to have a conversion to a mini-laparotomy because of too many adhesions. The quantity of ascites varied from 1.6 L to 5.0 L. Of the 17 patients with ascites, 88% had straw coloured ascites and 12% had yellowish-green coloured ascites.

**Table 3:** Laparoscopic findings in patients with definite or presumptive abdominal TB

Finding	Number	CD4 count(s)
Ascites, thick fibrous sheet covering lever, irregular peritoneum and tubercles covering bowel	1	7
Fibroadhesive peritonitis with tubercles covering bowel, omentum, and peritoneum	3	14,48
Ascites, fibroadhesive peritonitis, mesenteric lymph nodes, and tubercles covering small bowel and omentum	1	20
Fibroadhesive peritonitis with irregular peritoneum and matted bowel in ileo-cecal region	1	35
Ascites and white liver ulcerations	1	35
Fibrous stranding in the ileo-cecal region and granulation and ulceration covering spleen	1	41
Ascites, fibroadhesive peritonitis with a mass in ileo-cecal region and tubercles covering liver and peritoneum	1	47
Ascites, a retroperitoneal mass, and tubercles covering the bowel	2	49
Fibroadhesive peritonitis and mesenteric lymph nodes	1	49
Fibrous stranding around liver covered with a few tubercles	1	92
Ascites, fibroadhesive peritonitis and tubercles on bowel	1	103
Fibroadhesive peritonitis with ascites	1	128
Ascites with tubercles covering omentum and liver	1	139
Fibrous stranding around pale liver with small yellow ulcerations	1	214
Ascites, discoloured liver and caseating mesenteric lymph nodes	1	490
Ascites with tubercles covering the parietal peritoneum	1	-
Ascites, peritoneum covered with pus and caseating mesenteric lymph nodes	1	-

- indicates missing CD4 count

### Colonoscopy Findings

Of our three patients that had colonoscopies and were finally diagnosed with TB, one had a classical appearance of TB with nodular ulceration around the ileo-caecal valve. The second had non-specific inflammation in the cecum. The third had normal mucosa throughout.

## Discussion

The dual epidemic of HIV and tuberculosis is probably the greatest clinical challenge facing health workers in sub-Saharan Africa. Current HIV seroprevalence in Lusaka is probably close to the 2002 estimate of 22% such that a huge pool of at-risk persons is fuelling the epidemic of dual infection [19]. It has been known for many years that increasing immunodeficiency changes the clinical presentation so that extrapulmonary TB comes to dominate the clinical picture; abdominal TB is an important manifestation of extrapulmonary TB [20,21].

Defining the incidence or prevalence of abdominal TB is fraught with difficulties, and among these is the pressure to implement presumptive treatment to save lives. This was a major limitation of our study as a large proportion of patients fulfilling our inclusion criteria could not be studied because presumptive treatment invalidates culture as a diagnostic tool. We cannot therefore estimate the frequency of abdominal TB with precision, but our data suggest that a large and under-diagnosed problem exists amongst HIV positive adults in Zambia. If the 31 patients studied are representative of the 140 meeting our clinical criteria, then 99 (22/31 of 140) would have had abdominal TB. A survey in UTH of HIV seroprevalence in 2006 suggested that 73% of 201 medical inpatients were HIV-infected (Nzali Kancheya and Atia Jordan, unpublished data), so abdominal TB may be a larger problem than recognised previously. Of the 22 patients whom we found to have abdominal TB, this diagnosis was not being actively pursued by the admitting physicians in the majority, but as our study reached completion, the diagnosis of abdominal TB came to be considered more often among admitting physicians.

Those affected ranged in age from 18 to 46 years and there was a predominance of women over men. Our study shows that abdominal TB can affect single organs as well as multiple organs and can manifest itself in various ways. In contrast to a previous study [8], ascites was the most common manifestation in these HIV positive patients. Although it has been described [18], we did not find any ultrasound evidence of pancreatic involvement, but we did not measure serum amylase or lipase.

It appears that abdominal TB in Zambia occurs largely in people with CD4 cell counts of ≤150 cells/μL [22]. In a study from Brazil, the mean CD4 cell count of patients with extrapulmonary TB was 184/μL [21], but in our series the median was 49/μL. This could be a genuine biological difference or could be attributable to our patients having more advanced disease at presentation. This may reflect the poor state of Zambian health services which are struggling to cope with a massive burden of HIV-related pathology. We could identify no clear correlation between CD4 count and laparoscopic findings with our sample size.

It is important in a setting such as ours, with limited resources and high HIV and TB con-infection rates, for physicians to have a high clinical suspicion of abdominal TB. Due to the difficulties in differentiating its non-specific presenting features and difficulty of diagnosis, we aimed to create and test a useful algorithm. We found that 67% of patients that fitted our algorithm had abdominal TB, suggesting that fever and weight loss with accompanying abdominal symptoms and/or signs is an effective clinical tool to identify persons requiring an abdominal TB work-up. However, we almost certainly missed patients with abdominal TB without fever, which in a recent series was not present in up to one third of patients [23]. The majority of adults with HIV-related diarrhoea do not have fever and would not have been recruited to our study. In those that do have both of these features we believe it is important to consider abdominal tuberculosis, especially if blood cultures are negative.

In patients with fever and weight loss, the most predictive diagnostic markers were ultrasound detection of ascites (PPV of 73%), hepatomegaly (80%), and/or fibrous stranding (86%). Although abdominal lymphadenopathy had only moderate predictive value (69%), it is important to note that no controls had lymphadenopathy, so this finding should be reason for further investigation in HIV positive patients. If, as we suspect, some of the 6 patients with no confirmed diagnosis actually had abdominal TB, the PPV would be under-estimated. This could have happened if inaccurate targeting of biopsies missed the best tissue for biopsy, or if the mycobacteria had been killed by patients having been on anti-tuberculous chemotherapy for longer than they said.

Ascites culture was only positive in 25% of patients with this clinical feature. Our result was lower than that found in other studies [11], but it should be noted that previous studies did not separate findings among HIV positive and HIV negative patients.

Table 3 shows the diverse range of diagnostic appearances with laparoscopy of abdominal TB, including TB solely affecting the liver, spleen, peritoneum, or bowel, as well as a combination of these organs. In other laparoscopic studies, abdominal TB is categorized in discrete presentations. Bhargava et al. divided peritoneal TB into three types [17], but our findings do not fit such a clean pattern. It is not clear whether this is due to findings being subjective or whether it is because all our patients were HIV positive. For our patients that underwent colonoscopy, we noted that it is possible for normal looking mucosa to be colonized by *M. tuberculosis*. In the patient with positive visual findings we noted that the appearance was similar to other descriptions [24], including nodular ulceration in the ileal-caecal region.

## Conclusion

Our recommendations are that people with fever and weight loss with the features we have described should undergo ultrasound examination. The presence of ascites (especially with fibrinous stranding), hepatomegaly, or lymphadenopathy indicates that TB is likely and in settings where laparoscopy and colonoscopy would be impractical, as in much of Africa, early presumptive treatment is warranted. Lapatoromy may be necessary for those who do not respond to presumptive treatment. Ultrasound can also virtually rule out alternative diagnoses such as hepatic abscess, and abdominal lymphadenopathy is much more commonly due to abdominal TB than lymphoma. The most important point we can make is to keep a high index of suspicion for this difficult diagnosis as TB is treatable. If this simple message can be brought to the attention of primary care services we might be able to evaluate patients at an earlier stage of disease. The fact that 11 patients died waiting for treatment underlines the imperative to commence treatment promptly when clinical suspicion is high.

## Abbreviations

TB: tuberculosis; HIV: human immunodeficiency virus; CD4: cluster differentiation antigen 4+ cell; UTH: University Teaching Hospital; ZN: Ziehl-Neelsen; PAS: Periodic Acid Schiff stain; MGIT: mycobacterial growth indicator tube; ATCC: American type culture collection; FACS: fluorescence activated cell sorting; PPV: positive predictive value; NPV: negative predictive value.

## Competing interests

The authors declare that they have no competing interests.

## Authors' contributions

SG, ES, IZ, LZ, SHV, and PK designed the study, and SG, ES, LZ, VM and PK carried out the data collection. All authors were involved in the analysis and interpretation of the data, and contributed to the writing with ES, SG and PK responsible for most of the text.

## Pre-publication history

The pre-publication history for this paper can be accessed here:

http://www.biomedcentral.com/1471-2334/9/44/prepub
